# Primary screening for cervical cancer precursors by the combined use of liquid-based cytology, computer-assisted cytology and HPV DNA testing

**DOI:** 10.1038/sj.bjc.6600073

**Published:** 2002-02-01

**Authors:** P Vassilakos, P Petignat, M Boulvain, A Campana

**Affiliations:** Institute of Pathology, Geneva, Switzerland; Department of Gynaecology and Obstetrics, University Hospitals of Geneva, 1211 Geneva 14, Switzerland

**Keywords:** liquid-based cytology, computer-assisted cytology, Pap smear, human papillomavirus (HPV)

## Abstract

Primary screening for cervical cancer precursors has considerably evolved with the introduction of new technology to improve the early detection of disease. The objective of this study was to elaborate a diagnostic pathway integrating liquid-based and computer-assisted cytology and human papillomavirus DNA testing to focus screening on women at risk which may be more cost-effective for the healthcare system. A single laboratory analysis was conducted during a 5-month period using liquid-based cytology followed by human papillomavirus DNA testing for women with an abnormal result or with previous abnormal cytology. Human papillomavirus prevalence was estimated by testing 909 consecutive unselected samples. All slides were then rescreened using automated cytologic testing and triaged into a high- or low-score group according to computer results. Of the 8676 slides scanned, 352 had a test result of atypical squamous cells of undetermined significance or worse. Two hundred and ninety-seven (84.3%) samples with an atypical squamous cells of undetermined significance or worse result and 100% of those with detection of high-grade squamous intraepithelial lesions and carcinomas (HSIL+) were triaged into the high-score group. The combination of instrument scores and human papillomavirus results indicated that 51.0% of high score/human papillomavirus-positive cases should be considered as ASCUS+, while 99.6% of low-score/human papillomavirus negative cases remained negative in the final cytologic diagnosis, representing 49.0% of all cases**.** Of the screened women 89.5% should test negative for human papillomavirus and be reported as such in the final cytologic diagnosis. In conclusion, preliminary results suggest that this diagnostic pathway has the potential to improve primary cervical cancer screening and cost-effectiveness. By using a combination of testing methods to focus screening and clinical attention to cases at risk, it would be possible to lengthen screening intervals for 90% of women and to archive without further review all low-score/human papillomavirus-negative slides, representing 50% of the screening workload.

*British Journal of Cancer* (2002) **86**, 382–388. DOI: 10.1038/sj/bjc/6600073 www.bjcancer.com

© 2002 The Cancer Research Campaign

## 

Concern about the reliability of cervicovaginal cytology as a screening technique has been repeatedly voiced by both the public and health professionals in Europe and the United States. New technologies such as liquid-based cytology, automated computer-assisted screening, and human papillomavirus (HPV) DNA testing have enabled significant progress to be made in this important area of women's health.

Liquid-based, thin-layer cytology reduces sampling and preparation errors of the conventional smear. It has demonstrated improved specimen adequacy and sensitivity for the detection of cervical cancer and its cytologic precursors, squamous intraepithelial lesions (SILs) (Vassilakos *et al*, 2000), while providing less equivocal results and has led to 10% fewer recalls for repeat testing (Vassilakos, 1998a). Screening time of a thin-layer slide is also considerably shorter compared with the conventional smear (Vassilakos, 1998).

The AutoCyte PREP system (Tripath Imaging Inc, Burlington, NC, USA) is an automated screening tool for thin-layer processing. Independent studies have shown it to be significantly more effective for the detection of both low-grade squamous intraepithelial lesions (LSILs) and more severe diagnoses (Marino and Fremont-Smith, 2001; Vassilakos *et al*, 2000).

To further reduce screening or interpretation errors, automated devices were introduced as primary screeners or quality control instruments. The AutoPap system (Tripath Imaging inc, Burlington, NC, USA) addresses both sources of error. It has shown both superior sensitivity and specificity in the identification of all cervical abnormalities including atypical squamous cells of undetermined signficance (ASCUS) and LSILs alone or higher, when compared to current laboratory practice (Wilbur *et al*, 1998). A recent study found that the AutoPap system demonstrates the same capacities to detect and score abnormal liquid-based thin-layer slides as it does for abnormal conventional smears (Vassilakos *et al*, 2001).

Detection of high-risk HPV (HR-HPV) types by the Hybrid Capture II system (Digene Corporation, Gaithersburg, MD, USA) is of diagnostic and prognostic value. Recent studies show that HR-HPV types are strongly associated with SIL severity and cervical cancer (Bosch *et al*, 1995; Nobbenhuis *et al*, 1999; Walboomers *et al*, 1999). Persistence of these types is a necessary factor for the development of SIL and the subsequent progression to cancer (Nobbenhuis *et al*, 1999; Walboomers *et al*, 1999).

The majority of SILs without a HR-HPV type regress and minor cervical abnormalities such as ASCUS or LSIL in a HPV-negative patient are more likely to be false-positive cytologic diagnoses (Sherman *et al*, 1994; Cox *et al*, 1995). In addition, recent data indicate that HPV testing may be used efficiently in the management of ASCUS cases by referring to colposcopy only those women who test positive (Vassilakos *et al*, 1998; Solomon *et al*, 2001). Used as an adjunct to conventional or liquid-based cytology, HPV DNA testing has the potential to increase the sensitivity and specificity of primary screening (Cox *et al*, 1995; Ferenczy *et al*, 1996; Vassilakos *et al*, 1998). The high negative predictive value of the combined test, close to 100%, may improve cost-effectiveness by permitting screening intervals to be lengthened (Cuzick *et al*, 1999).

It can be postulated that a combined use of these three technologies could help to obtain improved levels of prognostic information and accuracy. The objective of our pilot study was to design a diagnostic pathway for a new screening option and patient management protocol so as to better focus attention to women who are at risk of having significant cervical abnormalities.

## MATERIALS AND METHODS

Cohort study participants were 8676 women who had a routine cervical Pap examination in Geneva, Switzerland, between September 2000 and January 2001, a population already studied with a low incidence of cervical cancer (Vassilakos *et al*, 2002). No selection criteria were applied to the cohort. Liquid-based samples were obtained from 33 gynaecologists of the region for routine screening purposes. A subset of 10% of specimens was created to determine the prevalence of HPV infection in the study population. Table 1

**Table 1:**
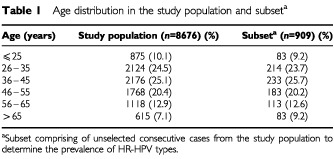
Age distribution in the study population and subset^a^

shows the age distribution in both the cohort and subset. No other socio-demographic data were collected. The study design is summarized in Figure 1

**Figure 1:**
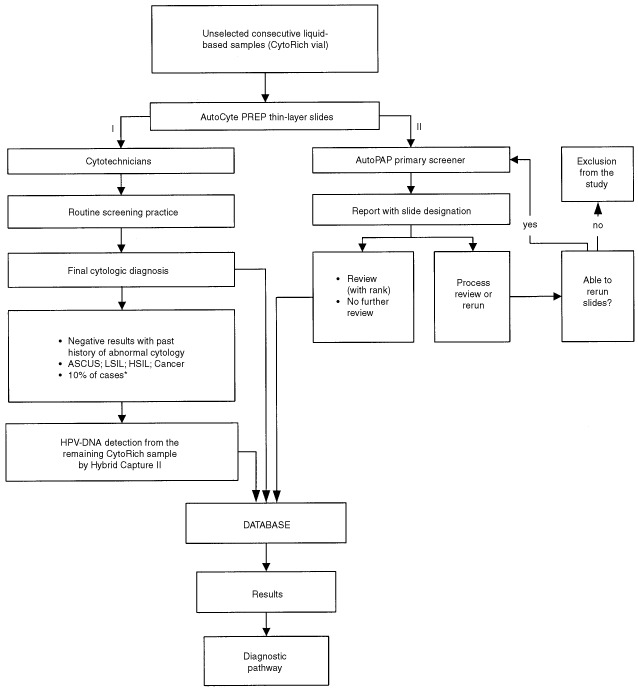
Study design. ASCUS=atypical squamous cells of undetermined significance; LSIL=low-grade squamous intraepithelial lesion; HSIL=high-grade squamous intraepithelial lesion; Cancer=squamous cell or other invasive carcinoma. *Subset composed by unselected consecutive cases from the cohort study population to determine HR-HPV types prevalence.

. Women were informed of the purpose of the study by their gynaecologist and gave their consent to use residual material from their samples.

### AutocytePrep cytology and interpretation

Within 48 h of collection, a thin-layer cervicovaginal slide was prepared using the robotic AutoCyte PREP system according to the manufacturer's instructions. In brief, the method uses a 24% ethanol-based preservative and the processing steps include vortexing, density gradient enrichment by centrifugation, decanting and resuspension of cell pellets followed by gravity sedimentation onto a cationically-coated slide, and automated Papanicolaou staining. All slides were initially processed according to the routine screening procedure of the Institute of Pathology, Geneva (Figure 1, step I).

Suspicious cases and those with a history of previous abnormal cytology were reviewed by a senior cytotechnician and forwarded to the cytopathologist (P Vassilakos) for final diagnosis. Final case diagnoses were classified according to the Bethesda nomenclature system and were considered as ‘reference diagnoses’.

### HPV DNA testing

Following preparation of the AutocytePrep slides, the remaining samples were used, according to our usual procedure, to conduct HPV DNA detection in patients reported positive for ASCUS or atypical glandular cells of undetermined significance (AGUS), or worse. Patients with normal results but with a previous history of abnormal cytology were also tested. The prevalence of HPV infection in the study population was estimated by testing a subset of 10% of unselected consecutive cases.

HPV testing was performed on a routine basis using the Hybrid Capture II assay. Each centrifuge tube contained 0.8 ml of residual cervical cells in water and was centrifuged at 800±15 *g* for 10±1 min. The supernatant was decanted and the residual removed by blotting the inverted tube on clean absorbent paper. Specimen Transport Medium (STM, Digene) (200 μl) was added to each cervical cell pellet and the tube vortexed for 15 s to resuspend the pellet. Denaturation reagent of 100 μl was then added to produce single-stranded DNA reacting with 13 full-length RNA probes recognizing HR-HPV types 16, 18, 31, 33, 35, 39, 45, 51, 52, 56, 58, 59 and 68, vortexed for 5 s, and incubated in a 65±2°C water bath for 90±5 min. The denatured specimen was then immediately tested by using the Hybrid Capture II assay according to manufacturer's instructions. A test was considered positive for the presence of HR-HPV-DNA if the relative light unit (RLU) obtained from the luminometer equalled to or exceeded the mean of the three positive control values (cut-off value equal to 1.0 pg ml^−1^). A RLU measurement <1.0 pg ml^−1^ indicated either the absence of the 13 HR-HPV types or HPV DNA levels below the threshold of detection.

### AutoPap classification scores

AutocytePrep slides were then subjected to the AutoPap, an automated primary screener (Figure 1, step II). A report was generated and each slide, based on the device assessment, was triaged into the following categories: ‘no further review’ (NFR), ‘review’ (R), ‘process review’ (PR), or ‘rerun’. A slide designated as NFR has a low probability of abnormality, and an R slide has a higher likelihood according to its ranking on a scale of 1 to 5, an aggregate measurement of numerous cytologic parameters. The higher the assigned score, the more likely a smear contains abnormalities and the lower the assigned score, the more likely a smear is negative. Each slide was also assigned an individual rank score expressed as a fraction and as a percentage. A high-score group was defined by including the 1st, 2nd and 3rd rank, and a low-score group by including the 4th, 5th and NFR category.

### Data analyses

All the results were entered into a Microsoft Access database (Redmond, Washington, USA) for analysis and elaboration of the screening and management diagnostic pathway (Figure 1). Trends were assessed with the chi-square test.

## RESULTS

From September 2000 to January 2001, a total of 9665 specimens were collected and initial screening was conducted using the AutoPap system. Of these, 8688 (89.9%) qualified for scanning and 977 (10.1%) were classified as PR and were excluded from the study because rerun was impossible. Nine hundred and sixty-five were cytologically negative and 12 were positive (one HSIL, five LSIL and six ASCUS). HPV DNA testing could not be performed for 12 positive cases (one AGUS, nine LSIL, two HSIL) because the residual sample was considered to be unsatisfactory or the clinician did not propose an HPV test. Finally, 8676 cases were included in the study.

The age-group distribution of the subset of 909 cases to estimate the prevalence of HPV was similar to the entire study population (Table 1). A prevalence of 9.5% (87 out of 909) for HR-HPV types was found. Following a further breakdown of HPV positives into high-score and low-score groups, the prevalence for each group was 9.8% (44 out of 447) and 9.3% (43 out of 462), respectively. We assumed that the prevalence for the total population screened should be distributed similarly. Table 2

**Table 2:**
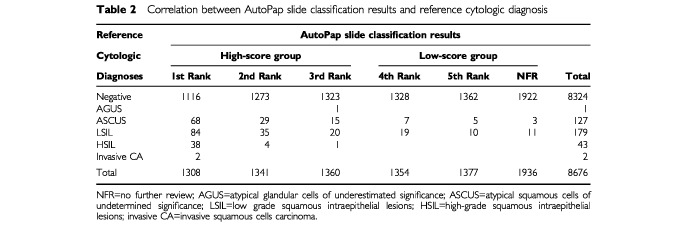
Correlation between AutoPap slide classification results and reference cytologic diagnosis

presents the correlation between AutoPap slide classification results and cytologic diagnosis. Figure 2

**Figure 2:**
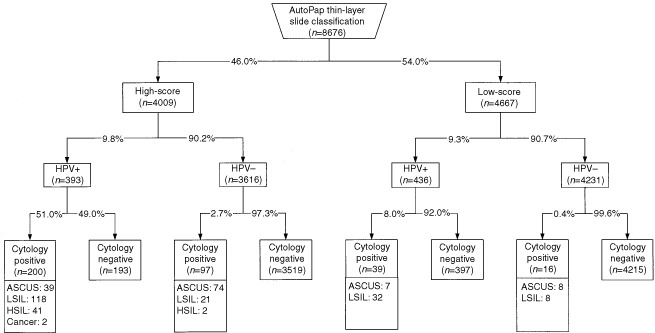
Combined results of manual screening, automated screening, and HPV-DNA testing. ASCUS=atypical squamous cells of undetermined significance; LSIL=low-grade squamous intraepithelial lesion, HSIL=high-grade squamous intraepithelial lesion; Cancer=invasive squamous cell carcinoma; HPV=human papillomavirus; HPV+=positive test for HR-HPV types; HPV−=negative test for HR-HPV types.

summarizes results according to the combination of AutoPap scores, HPV testing, and final cytologic diagnoses. Of the 8676 slides scanned, 4009 (46.0%) were assigned to the high-score group and 4667 (54.0%) to the low-score group. The distribution according to the combined results is represented as follows: 51.0% of high-score/HPV-positive cases were diagnosed as ASCUS+, while 8% of low-score/HPV-positive cases were diagnosed as ASCUS+. High-score and low-score/HPV-negative cases were negative at manual screening in 97.3 and 99.6%, respectively.

An association was found between HPV positivity and cytologic diagnosis. Sixty-six out of 868 (7.6%) of cytologically-negative cases without a past history of abnormal cytology were HPV-positive. In contrast, 21.7% (63 out of 290) of cytologically-negative cases with a past history of abnormal cytology were HPV-positive. Finally, 35.9% (46 out of 128) of ASCUS, 83.7% (150 out of 179) of LSIL and 95.5% (43 out of 45) of HSIL+ were HPV-positive. Chi-square for trend were 575, *P*<0.001.

HR-HPV positivity and automated scoring were not closely related. This is not surprising since the Autopap system uses a statistical classifier algorithm to assign a numerical score to each slide. In the presence of benign cellular changes sharing some morphologic similarities with significant cervical abnormalities, the slide is triaged into the high-score group. These slides may be HR-HPV negative. On the other hand, HPV positivity is found in samples without cervical abnormalities; in these cases, the slide may be triaged into the low-score group.

## DISCUSSION

Although organized screening programs based on the Papanicolaou (Pap) smear have been very successful in reducing mortality, a major problem has emerged. Cervical cancer has not been eradicated and its incidence has remained virtually constant for several years. In Switzerland, cervical cancer is still among the 10 leading causes of cancer with approximately 400 new cases and 100 deaths annually, mostly occurring in women over 65-years-old (Ligue Suisse Contre le Cancer, 1998). The majority of cases have never had a Pap test, or have not had one in the previous 5 years.

However, false-negative results from Pap smears do occur, leading to deaths in routinely screened women. Results of one study showed that 14% of women with an invasive cervical cancer or HSIL had received a negative smear result within the 2 years prior to diagnosis. Approximately two-thirds of false-negative smears were related to sampling errors, and the remaining third were due to screening and/or interpretative errors, mainly due to the small number of diagnostic cells present in a suboptimal smear (Vassilakos, 1998b).

Studies carried out elsewhere have also confirmed that the Pap smear is not a highly effective primary screening test in the detection of cervical cancer and its precursors (Koss, 1989). It was found that 40% of cervical cancers occur in women who recently received negative test results (Slater *et al*, 1994), and that a single Pap test misses up to 50% of cervical cancer precursors (Federal Study, 1999). In England and Wales, 47% of the invasive cervical cancer found in women under the age of 70 years occur despite an apparently adequate screening history (Sasieni *et al*, 1996).

It has also been demonstrated that false-negative rates of less than 5% are unlikely to be achieved (Kreiger and Naryshkin, 1994) and may occur even when smears are taken by a gynaecologist under colposcopy (Beeby *et al*, 1993). Until a prophylactic HPV vaccine becomes available, screening will need to be continued and cervical cytology will necessarily be in a period of transition. Significant improvements are expected with the development and wider implementation of new technologies. In Switzerland, 60% of Pap tests are currently performed with liquid-based thin-layer technology and are reimbursed by the health insurance companies (survey of the Swiss Federal Office of Social Insurances). HPV DNA testing, determined from an aliquot of the same sample used for liquid-based cytology, is also widely used as a ‘reflex’ test to triage patients with ASCUS, thus eliminating the need for a repeat office visit.

It is reasonable to expect that the ultimate way forward in the next 5–10 years will be the use of standardized liquid-based cytologic preparations, automated screening, HPV DNA testing, early detection with molecular markers, or completely novel methods. These technologies can provide improved sensitivity and specificity but at increased cost. For this reason, the way in which we use them will define the future of cervical cancer prevention. We believe that they should be considered as complementary diagnostic tools and not as competing methods. Their combination has been shown to increase sensitivity and specificity (Ferenczy *et al*, 1996; Vassilakos *et al*, 1998) and this may allow more cost-effective screening strategies and improved patient management (Cuzick *et al*, 1999; Cuzick and Sasieni, 1999; Solomon *et al*, 2001).

**Figure 3:**
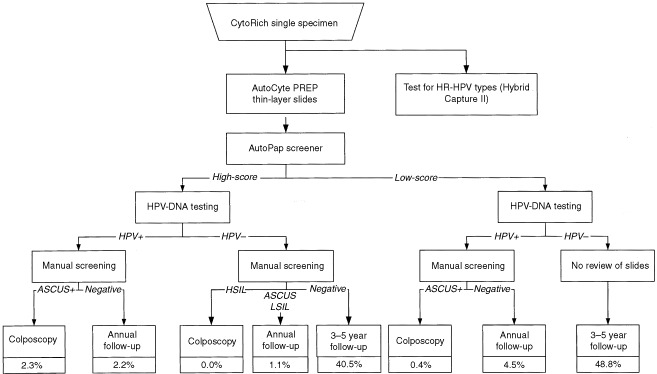
Proposed diagnostic pathway for cervical cancer detection using liquid-based thin-layer slides, computer-assisted screening, and HPV-DNA testing. ASCUS+=atypical squamous cells of undetermined significance, low-grade squamous intraepithelial lesion, high-grade squamous intraepithelial lesion, squamous cell or other carcinoma. HPV=human papillomavirus; HPV+=positive test for HR-HPV types; HPV−=negative test for HR-HPV types.

Our results demonstrate that by using combined technologies a new strategy can be introduced to screen and manage cervical cancer precursors (Figure 3

).

In common with other studies (Meijer *et al*, 1998; Cuzick and Sasieni, 1999), we estimated that approximately 90% of cases are double-negative screens (HPV-negative/cytology negative). The potential benefit of this result is that it provides a level of certainty to women that no significant epithelial abnormality has been missed. Furthermore, savings for the healthcare system may be achieved by safely lengthening the screening intervals (Cuzick and Sasieni, 1999). For cases consistently double-negative, screening may be conducted not only less frequently but even stopped, especially among older women.

Another positive effect of the proposed diagnostic pathway is the possibility to reduce laboratory costs related to manual screening. The latter could be limited to only high-score slides and those triaged into the low-score/HPV-positive group. Nearly 50% of slides (low-score/HPV-negative group) could then be archived without further review since virtually all of them are negative in the final cytologic diagnosis. In 0.4% of these slides, we found some minor grade abnormalities (eight ASCUS and eight LSIL) corresponding to a false-negative rate of 5.2%. This rate compares favourably with the false-negative rate for ASCUS and LSIL reported in routine practice. The detected abnormalities are considered to be related to a transient HPV infection or an inflammation and will regress spontaneously. It is also highly unlikely that an underlying HSIL+ lesion will be missed since all HSIL+ will be triaged into the high-score group. In our study, 95.5% of these cases were HPV-positive.

The largest volume of patients with abnormal cytology requiring focused clinical attention and triage concerns those identified with ASCUS and LSIL. In the context of an ASCUS cytologic diagnosis, the triage by HPV DNA testing is considered as a sensitive method for the detection of an underlying HSIL (Solomon *et al*, 2001). The high negative predictive value of the HPV testing may reduce patient anxiety, prevent overtreatment and potentially save costs by decreasing repeat visits for equivocal diagnoses. This can be achieved by adopting a conservative approach for those women with ASCUS or LSIL who tested negative for HPV.

Based on the results of our study, 2.7% of all screened women should be referred for immediate colposcopy, 7.8% should return for a yearly follow-up, and 89.5% could safely lengthen their screening interval to 3 or even 5 years instead of the yearly interval currently applied in Switzerland and elsewhere. The colposcopy referral rate compares favourably with current practice when the option of direct referral to colposcopy for all ASCUS+ cases is adopted.

The major advantage of this proposed combined screening option is the greater protection offered by focusing screening attention on cases who are at greater risk of having a significant cervical abnormality. In our study population 84.3% of ASCUS+ cases and 100% HSIL+ cases were triaged into the high-score group. Among these, 57.0% of ASCUS+ and 95.5% of HSIL+ were HPV-positive. Importantly, one out of two cases assigned to the high-score/HPV-positive group was reported as ASCUS or worse in the final cytologic diagnosis. A quality control rescreening may be applied for high-score/HPV-positive slides reported as negative by the cytotechnologists. It can be expected that this measure will enhance the false-negative detection because it is efficiently targeted. It may be also more cost-effective compared with present practice since less than 3% of all screened slides will be reviewed instead of the current 10% random slide selection process. The progressive rate of HPV positivity related to cervical lesions can also be used as a quality control of cytopathologic and histopathologic expertise, particularly concerning excessive cytological diagnoses of minor cervical abnormalities (ASCUS or LSIL). Failure to detect the accepted rates would suggest that the Bethesda classification criteria are incorrectly applied.

The ability of this diagnostic pathway to identify cases with a high probability of abnormality and those that can be archived without further review will assist cytotechnologists to screen more accurately slides designated for review. In addition, the tedious part of manual screening and associated tiredness that occurs when predominantly normal slides are screened will be significantly reduced since attention is focused to cases at risk.

A problem that may emerge with the introduction of the new approach is the long-term surveillance of the HPV-positive/ cytology negative cases. We estimate that it will concern approximately 7% of the screened women in our population. The clinician caring for the patient may be concerned by the sexual transmission of the HPV infection or of leaving untreated an underlying significant lesion, and this may lead to an increase in the number of colposcopies. We consider that the availability of more accurate information on this topic to women and the clinician would ensure that both would be more at ease with an annual follow-up only. It should be emphasized that HPV is a very common virus and for most women the infection is transient and very unlikely to progress to cancer. Two HPV tests 8 to 12 months apart are sufficient to allow clearance of infection. Only persistence of certain types is a risk factor and this risk can be eliminated by participating in regular recommended cervical screening programmes. The major concern of our cervical cancer screening approach is the impact on the cost-effectiveness of this combination of new technologies. We are presently conducting a study to evaluate this aspect in detail. Substantial savings are expected to arise from the extended screening interval, the reduced rate of recalls, the 50% rate of archived slides requiring no further review from the cost-effective oriented quality control and from the reduced screening time of those slides requiring manual review. Savings in terms of absence from work and travel for unnecessary repeated tests and treatments, as well as in terms of creating unnecessary anxiety to women, need also to be evaluated. The issue of the age at which to start combined screening tests is another important component of a successful and cost-effective system (Cuzick *et al*, 1999). All the benefits stated above, together with an increased test sensitivity, could offset the increased cost of the technology.

In conclusion, our study indicates that a diagnostic pathway combining new technologies is realistic and has the potential to improve cervical cancer screening. This approach could be cost-effective for the patient and clinical practice as it focuses screening and clinical attention to cases at risk, thus allowing to effectively test and treat women for significant cervical abnormalities. Healthcare costs could be reduced by lengthening screening intervals to 90% of women and by archiving without further review 50% of the slides. However, additional clinical studies are necessary to validate these preliminary results and to assess the cost-impact of introducing new technologies.
